# Temporal dynamics of teleost populations during the Pleistocene: a report from publicly available genome data

**DOI:** 10.1186/s12864-021-07816-7

**Published:** 2021-06-30

**Authors:** Jia Li, Chao Bian, Yunhai Yi, Hui Yu, Xinxin You, Qiong Shi

**Affiliations:** 1 Shenzhen Key Lab of Marine Genomics, Guangdong Provincial Key Lab of Molecular Breeding in Marine Economic Animals, BGI Academy of Marine Sciences, BGI Marine, BGI, Shenzhen, Guangdong China; 2 Center of Reproduction, Development and Aging, Faculty of Health Sciences, University of Macau, Macau, China; 3 BGI Education Center, University of Chinese Academy of Sciences, Shenzhen, Guangdong China; 4 Laboratory of Aquatic Genomics, College of Life Sciences and Oceanography, Shenzhen University, Shenzhen, Guangdong China

**Keywords:** Global climate change, Temporal dynamics, Effective population size, High-quality genome assembly, Teleost

## Abstract

**Background:**

Global climate oscillation, as a selection dynamic, is an ecologically important element resulting in global biodiversity. During the glacial geological periods, most organisms suffered detrimental selection pressures (such as food shortage and habitat loss) and went through population declines. However, during the mild interglacial periods, many species re-flourished. These temporal dynamics of effective population sizes (*N*_*e*_) provide essential information for understanding and predicting evolutionary outcomes during historical and ongoing global climate changes.

**Results:**

Using high-quality genome assemblies and corresponding sequencing data, we applied the Pairwise Sequentially Markovian Coalescent (PSMC) method to quantify *N*_*e*_ changes of twelve representative teleost species from approximately 10 million years ago (mya) to 10 thousand years ago (kya). These results revealed multiple rounds of population contraction and expansion in most of the examined teleost species during the Neogene and the Quaternary periods. We observed that 83% (10/12) of the examined teleosts had experienced a drastic decline in *N*_*e*_ before the last glacial period (LGP, 110–12 kya), slightly earlier than the reported pattern of *N*_*e*_ changes in 38 avian species. In comparison with the peaks, almost all of the examined teleosts maintained long-term lower *N*_*e*_ values during the last few million years. This is consistent with increasingly dramatic glaciation during this period.

**Conclusion:**

In summary, these findings provide a more comprehensive understanding of the historical *N*_*e*_ changes in teleosts. Results presented here could lead to the development of appropriate strategies to protect species in light of ongoing global climate changes.

**Supplementary Information:**

The online version contains supplementary material available at 10.1186/s12864-021-07816-7.

## Background

Global climate oscillation is an ecologically important element leading to the global biodiversity. Global climate fluctuations can exert a wide range of selection pressures to influence the distribution and proliferation of organisms [1, 2]. In earth’s history there had been five mass extinctions [3], several of which were related to climate change. For example, possible causes for the Ordovician-Silurian extinction events (443.8 mya) include glaciation and volcanism [4]. Moreover, detrimental environmental changes associated with glaciation might have also contributed to this extinction. First, the cooling global climate may have elicited the resident species to an intense greenhouse extinction [5, 6]. Secondly, a reduction in the sea level drained vast epicontinental seaways, most likely destroying the habitats of many endemic communities [7–9]. Volcanism released harmful gases and ashes into the atmosphere, thereby causing greenhouse effect, atmosphere darkening, reduction in photosynthesis and atmospheric oxygen, as well as destruction of critical food webs [10]. Nearly all major taxonomic groups on earth were affected during the Ordovician-Silurian extinction event, eliminating 49–60% of marine genera and nearly 85% of marine species [11, 12].

Almost all species are influenced by climate changes and many have developed various responses, such as transference of habitat and alterations in phenotype and genotype [13, 14]. Climatic fluctuations in earth’s past dramatically impacted the demography and distribution of various species. During the glaciation periods, native environmental conditions were destroyed and replaced with ice and permafrost. Consequently, the cooling global climate produced either species extinction or forced species to migrate into new habitats. During the interglacial periods, however, ice and permafrost melted, which provided more suitable habitats for diverse species to recolonize [15, 16].

How to infer the historic changes in effective population size (*N*_*e*_) has long plagued ambitious researchers. Several practical approaches, such as the Skyline-Plot Methods [17], have been developed to reconstruct demographic history. However, most of these methods only employ a restricted number of marker loci [18, 19], or due to heavy computation requirements they cannot be used for large-scale datasets [20, 21]. With the rapid development of high-throughput sequencing technologies, scientists have generated massive genomic data and maker loci for many target species. This advantageous development, and several other new analytical approaches, have enabled the use of large numbers of loci to infer the changes of *N*_*e*_. For instance, the pairwise sequential Markovian Coalescent (PSMC) method establishes a model within a single diploid genome inferring the *N*_*e*_ as well as the projected time of the most recent common ancestor (TMRCA [22];). Compared with other methods, PSMC works effectively on resequencing data too. PSMC method produces less potential ascertainment bias and is easier to implement. Unlike other methods with simulations of predefined models (such as parametric structure of times, divergences, and size changes), the PSMC method inferring the *N*_*e*_ for each species does not require any predefined model.

Many genome projects have sought to investigate the relationship between sea levels and the population changes of amphibious fishes [23]. In addition, it has been a focus of research to detect decline or expansion of bird population during the Quaternary period [24, 25]. These studies have attempted to analyze the interaction of population size with geological events [26–28]. Nadachowska-Brzyska et al. [24] explored historic population changes in many avian species. For example, it was found that the population size of endangered birds experienced long-term decline. During the Quaternary period, the fluctuation in population size presented consistent correlative characteristics for many bird species. Both extinction and speciation, in fact, coexisted in this period.

However, cold-blooded fishes are more vulnerable than warm-blooded birds to climate changes [29]. Fish metabolic rates are closely linked to body temperature, which are dependent on external temperature conditions [30]. Changes in fish population size are therefore more strongly correlated with climate fluctuations than those observed in birds.

In this paper, the PSMC method was employed to infer population size changes in twelve representative fish species (Table 1), starting from 10 kya up to 10 mya. It was found that multiple rounds of *N*_*e*_ increase and decline occurred in most of the examined fish species. This was predicted to be directly generated by the drastic oscillations in global climate during the Neogene and the Quaternary periods. Ten of the twelve studied fishes had experienced drastic reductions in *N*_*e*_ since the beginning of the last glacial period (LGP, 110–12 kya) [31, 32], which was slightly earlier than the pattern observed in birds [24].

**Table 1:** Summary of the examined teleost species and their genomes

Common name	Scientific name	Assembly version	Genome size (bp)	Gene number
Zebrafish	*Danio rerio*	GRCz11	1,373,471,384	25,709
Mexican tetra	*Astyanax mexicanus*	GCA_000372685.1	1,191,242,572	23,041
Atlantic herring	*Clupea harengus*	GCA_000966335.1	807,711,962	23,095
Japanese flounder	*Paralichthys olivaceus*	GCA_001904815.2	545,775,252	21,787
Tongue sole	*Cynoglossus semilaevis*	GCA_000523025.1	470,199,494	21,381
Three-spined stickleback	*Gasterosteus aculeatus*	BROAD S1	461,533,448	20,772
Channel catfish (BGI)^a^	*Ictalurus punctatus*	PRJNA319455	845,391,728	21,937
Large yellow croaker	*Larimichthys crocea*	GCA_000972845.1	678,938,134	24,418
Asian seabass	*Lates calcarifer*	GCA_001640805.1	668,481,366	24,348
Nile tilapia	*Oreochromis niloticus*	GCA_000188235.1	927,383,394	21,437
Asia arowana^b^	*Scleropages formosus*	GCA_001624265.1(Golden)	777,359,276	22,016
		GCA_001624245.1(Green)	746,544,453	21,524
		GCA_001624255.1 (Red)	738,407,480	21,256
Spotted green pufferfish	*Tetraodon nigroviridis*	TETRAODON 8.0	358,618,246	19,583
Fugu	*Takifugu rubripes*	FUGU5	358,618,246	18,505
Grass carp^c^	*Ctenopharyngodon idella*	–	1,076,149,922	32,785
Atlantic salmon	*Salmo salar*	GCA_000233375.4	2,966,890,203	43,899
Atlantic cod	*Gadus morhua*	gadMor1	832,114,588	20,083
Medaka	*Oryzias latipes*	ASM223467v1	734,057,086	19,669
Amazon molly	*Poecilia formosa*	GCA_000485575.1	748,923,461	23,613
Southern platyfish	*Xiphophorus maculatus*	GCA_000241075.1	729,662,853	20,367
Spotted gar	*Lepisosteus oculatus*	GCA_000242695.1	945,878,036	18,328
Coelacanth	*Latimeria chalumnae*	GCA_000225785.1	2,860,591,921	19,555
Tropical clawed frog^d^	*Xenopus tropicalis*	GCA_000004195.1	1,511,735,326	18,442

^a^The high-quality genome assembly of the Chinese channel catfish population is available in GigaScience Database at http://dx/doi.org/10.5524/100212.

^b^The genome information of golden arowana was used for the divergence time evaluation.

^c^The genome assembly of grass carp was downloaded from the Grass Carp Genome project at http://www.ncgr.ac.cn/grasscarp/.

^d^The outgroup for construction of the divergence time tree (see Figure S1).

## Results

### Effective population sizes of representative fishes during the quaternary period

**Fig. 1:**
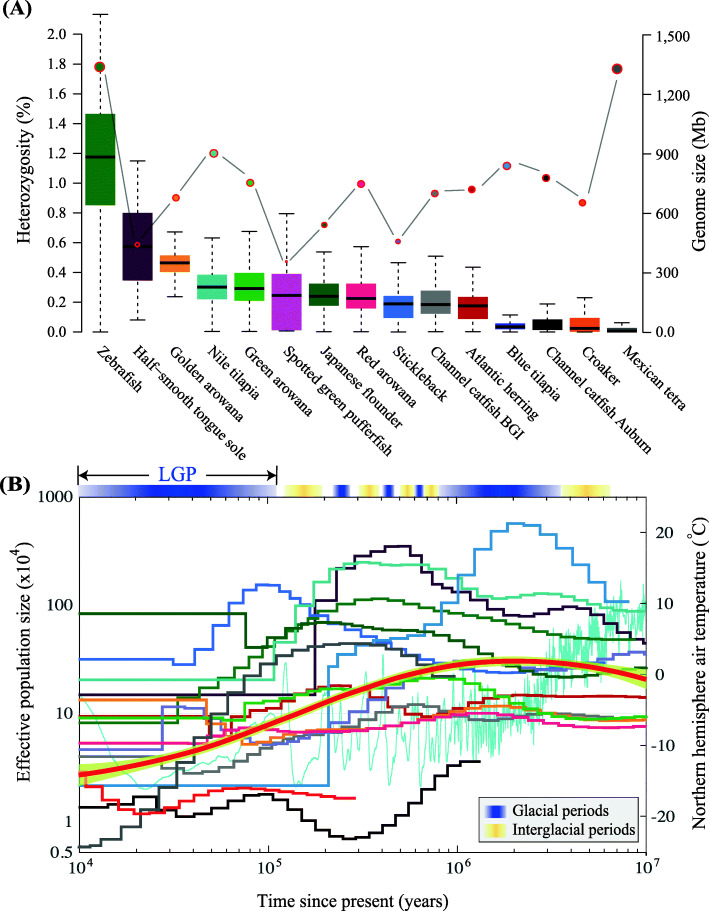
Demographic history and genome-wide heterozygosity of 12 representative fishes with high-quality genome assemblies. **A** The boxplot represents the genome-wide heterozygosity that was estimated from genomic 500-kb windows. Genome sizes of the examined fish species were presented by dots. **B** Historical *N*_*e*_ of the 15 examined fish genomes. The x axis depicts the historical time, and the y axis represents *N*_*e*_. These plots were scaled using species-specific mutation rates (u) and generation times (g; Table S1). The color of each species is consistent with that of boxplot in (**a**). Gradient blue bars above the plots represent the glacial periods and the gradient yellow bars represent the interglacial periods. The red smooth line shows the best-fit model with 95% confidence interval. LGP, the last glacial period (110–12 kya)

In the present study, genome-wide heterozygosity varied among the twelve representative fish species with high-quality genome assemblies (Fig. 1A). Interestingly, the mean heterozygous sites per nucleotide in the genome sequence of zebrafish (*Danio rerio*) was 1.1 × 10^− 2^. This number is much higher than those in other fishes. The zebrafish variant was directly obtained from the Ensembl database, which integrated comprehensive results based on data from different individuals. Hence, this guaranteed the higher sequencing coverage and efficient variant data in zebrafish, possibly contributing to the higher heterozygosity.

The heterozygous rate per nucleotide in Mexican tetra (*Astyanax mexicanus*) was about 2 × 10^− 4^, representing the lowest among these examined fish species. The mean heterozygous rates observed among the three varieties of Asian arowana (*Scleropages formosus*) were 4.5 × 10^− 3^, 2.7 × 10^− 3^ and 2.0 × 10^− 3^ (golden, green and red arowana), respectively. The rate was 2.0 × 10^− 3^ for Japanese flounder (*Paralichthys olivaeus*), which was greatly reduced compared with the heterozygosity in half-smooth tongue sole (*Cynoglossus semilaevis*; 5.3 × 10^− 3^).

The historical *N*_*e*_ values of the twelve representative fish species were inferred using the PSMC approach over a range from 10 kya up to 10 mya (Fig. 1B). The results allow us to track population changes during the Neogene period (2.58–23.03 mya) and the Quaternary period (following the Neogene Period spanning from 2.58 mya to present). Over these periods, the maximal *N*_*e*_ values among the twelve examined fish species had experienced remarkable variations, ranging from 2,000,000 to 6000,000 in blue tilapia (*Oreochromis aureus*), Nile tilapia (*Oreochromis niloticus*) and half-smooth tongue sole; the minimal *N*_*e*_ ranged between 1000 and 6000 (large yellow croaker *Larimichthys crocea* and Mexican tetra), 50,000–10,000 (Atlantic herring *Clupea harengus*, channel catfish *Ictalurus punctatus*, and spotted green pufferfish *Tetraodon nigroviridis*), and 100,000–200,000 (half-smooth tongue sole, green arowana, and golden arowana). Thus, the *N*_*e*_ values ranged in at least four orders of magnitude among various teleost species during the examined periods.

Ten of the twelve examined fish species demonstrated drastic reductions in *N*_*e*_ at the beginning of the LGP. This either occurred just before the LGP or at the beginning of this period (Fig. 1b). For example, the green arowana reduced from approximately 180,000 individuals at the beginning of the LGP to 9600 by the end of this period. Three-spined stickleback (*Gasterosteus aculeatus*) experienced a dramatic five-fold reduction from approximately 1,540,000 to 31,000 individuals during the same period.

### Fluctuations of effective population expansions and contractions

Eleven of the twelve examined fish species in our analysis showed that *Ne* had changed dramatically over the observation time. Fluctuations of effective population expansions and contractions had considered to be a general feature of most fishes.

**Fig. 2:**
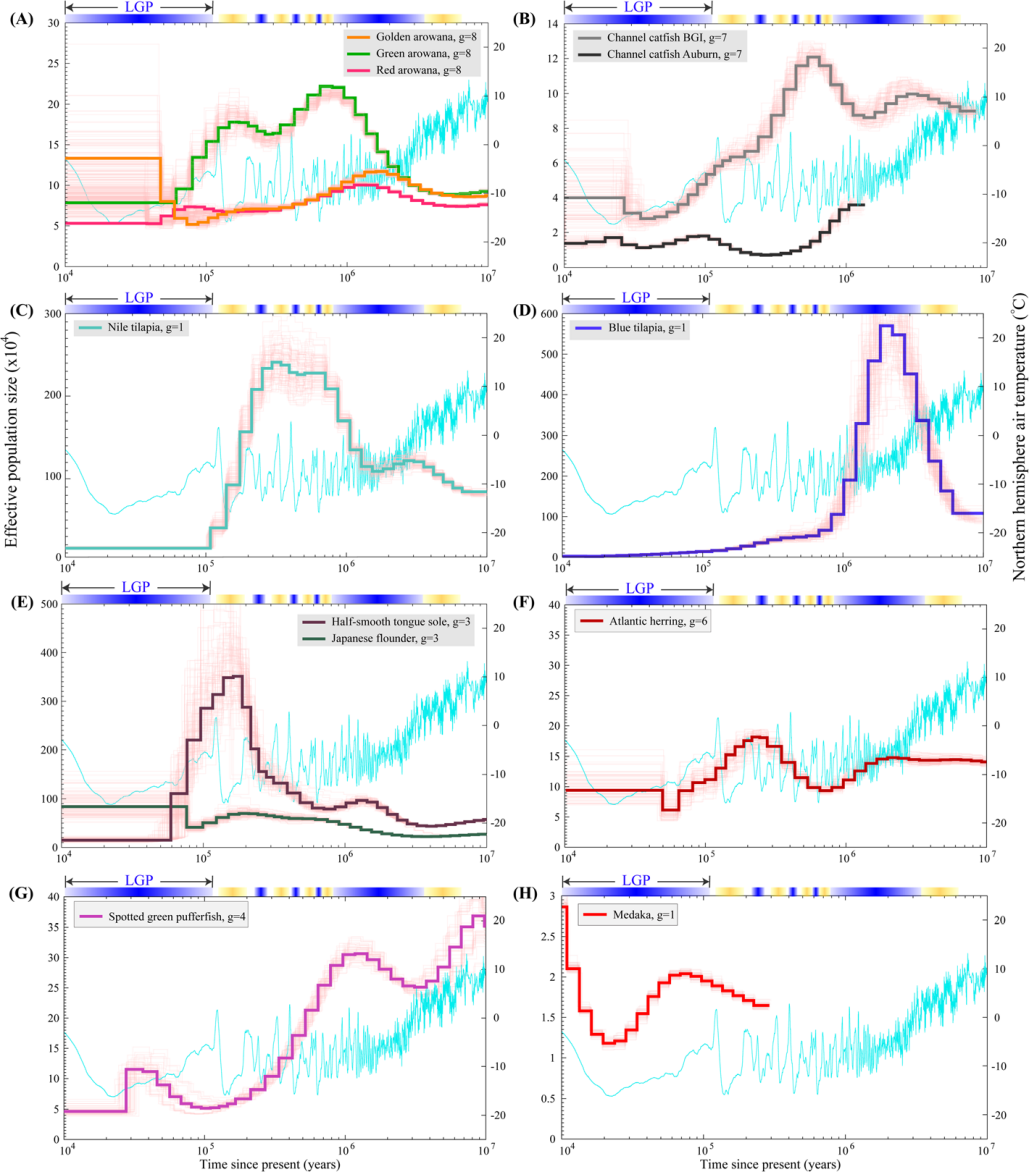
Fluctuations of Effective Population Expansions and Contractions. The pink curves represent PSMC estimates for 100 bootstrapped sequences. The light blue line indicates changes of the Northern hemisphere air temperature [32]. The gradient blue bars above the plots represent the glacial periods and the gradient yellow bars represent the interglacial periods. “g” is the generation time of each species. **A** Arowanas. **B** Catfishes. **C** Nile tilapia. **D** Blue tilapia. **E** Half-smooth tongue sole and Japanese flounder. **F** Atlantic herring. **G** Spotted green pufferfish. **H** Medaka

More specifically, the *N*_*e*_ among three varieties of Asian arowana varied remarkably (Fig. 2A). Before 2 mya, the three varieties presented relatively similar *N*_*e*_ values. Since then, however, the green arowana had higher *N*_*e*_ values (100,000–240,000) than the other two varieties, reaching the first peak (240,000) at 600–800 kya; after that, it experienced a reduction to 80,000 until approximately 60 kya. The red and the golden arowana demonstrated slight variations with *N*_*e*_ values of 50,000–100,000 until approximately the beginning of the LGP, when the red arowana experienced an undulation of rising steadily first and then falling to 50,000 until 50 kya; however, the golden arowana experienced an opposite trend simultaneously from 70,000 decreasing to around 50,000 and then rising again to 130,000 until 50 kya. At the end of the examined period (until 10 kya), the *N*_*e*_ of golden arowana was approximately three-fold greater than the red arowana and approximately two-fold greater than the green arowana. In our previous study [33], it was predicted that Asian arowana and spotted gar (*Lepisosteus oculatus*) diverged 384 mya. Since then, three varieties diverged approximately 1–4 mya to evolve as an independent lineage (Figure S1). Green arowana diverged from the other two varieties approximately 3.8 mya, and the latter two varieties diverged approximately 1.7 mya. The *Ne* variations could briefly reflect the evolutionary trajectories of this fish species. Prior to 2 mya, three varieties of arowana had similar population sizes with a slightly lower value in the red arowana, implying that these arowana varieties could belong to the same lineage before 2 mya. Subsequently, the green arowana diverged from the other two varieties and experienced a different evolutionary process, with a remarkable expansion in population (the first and second peaks). Although the red arowana diverged from the golden arowana approximately 1.7 mya, both varieties experienced similar changes in *N*_*e*_ from 1 mya.

In 2016, Liu et al. [34] completed the genome sequencing project of channel catfish (from an American population), and generated a draft reference genome assembly with a total length of 783 Mb and a scaffold N50 of 7.7 Mb. Subsequently, we reported another high-quality genome assembly of channel catfish (from a Chinese population; Chen et al., 2016), with approximately 845 Mb in total length and a scaffold N50 of 7.2 Mb (see Table 1). Although the Chinese population has been recognized as the introduction from America, genetic characteristics of the Chinese population seem to be significant different from the American counterpart after 30 years of artificial breeding in China [35]. These differences were also witnessed from the present prediction of the temporal dynamics in the *N*_*e*_. The similar trend of *N*_*e*_ was observed from 10 to 100 kya; prior to that, the tendencies of *N*_*e*_ for both populations experienced dramatic differences (see Fig. 2B). The heterozygosity in the Chinese population was 0.2%, which was higher than the American population (0.155%). These results confirmed the observed differences at a genomic level between the two populations.

Nile tilapia had a stable *N*_*e*_ (around 1000,000) until approximately 1 mya, when it increased to a peak of 2,500,000 around 300 kya. Subsequently, Nile tilapia experienced a drastic reduction in *N*_*e*_ to 200,000 within the LGP (see Fig. 2C). Recently, a high-quality genome assembly of another tilapia species, blue tilapia (*Oreochromis aureus*), was reported by us [36], which had a significant variation in population size compared to the Nile tilapia (Fig. 2D). More specifically, from 6 to 2 mya the *N*_*e*_ of the blue tilapia presented an approximately 6-fold increase (at the peak) around 2–3 mya, when it reached 5,700,000 at 2 mya. During the following 1 million years, the *N*_*e*_ of blue tilapia declined rapidly. Since then, the population size of blue tilapia has never recovered, but it was steadily declining to less than 200,000. The two sequenced tilapia species diverged approximately 23.2 mya [36], suggesting that they had experienced different evolutionary processes during the examined period. The population of blue tilapia flourished until the junction of the Neogene and Quaternary periods. However, blue tilapia experienced one cycle of population expansion and contraction (see Fig. 2D). Possibly due to the decline in temperature within the LGP and the low tolerance to low temperature, the two tilapias had low *N*_*e*_ values during the LGP (see more details in Fig. 2C and D).

The effective population size of two sequenced flatfish species, half-smooth tongue sole and Japanese flounder, also were fluctuating (Fig. 2E). Before the LGP, the two Pleuronectiformes species had similar trends of the *N*_*e*_, with a steady increase over time. Both flatfishes experienced a sharp decline since the beginning of LGP. The *N*_*e*_ of Japanese flounder reached its lowest level around 100 kya, which was 1 kya earlier than the half-smooth tongue sole. The most significant variance between the two flatfishes occurred 60 kya, when the population of the half-smooth tongue sole was maintained at the lowest level while the population of the Japanese flounder rapidly increased to high levels.

The population sizes varied significantly in Atlantic herring (Fig. 2F) and spotted green pufferfish (Fig. 2G) over time, especially within the LGP. The *N*_*e*_ values of these two fish reached peaks at 0.2–1 mya. Interestingly, the increase in populations was observed in these species during the LGP. Japanese medaka (*Oryzias latipes*; Fig. 2H) demonstrated *N*_*e*_ changes within a shorter period than the other species. We therefore propose that the low coverage of SNP data set (Supplementary Table S1) may contribute to this phenomenon.

### Other teleost species

**Fig. 3:**
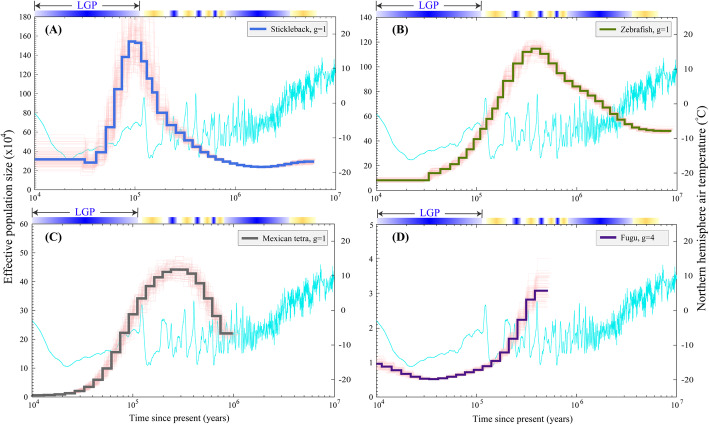
Temporal dynamics of effective population size for other teleost species. The pink curves in each sub-plot represent PSMC estimates for 100 bootstrapped sequences. The light blue line indicates changes of the Northern hemisphere air temperature [32]. The gradient blue bars above the plots represent the glacial periods and the gradient yellow bars represent the interglacial periods. “g” is the generation time of each species. **A** Stickleback. **B** Zebrafish. **C** Mexican tetra. **D** Fugu

Four fish species with a shorter generation time, including stickleback, zebrafish, Mexican tetra and fugu (*Takifugu rubripes*), presented similar population trends (Fig. 3). Each species showed a population rise at first and then a decline with differing times to reach peak size. The peak population of stickleback (Fig. 3A) reached 1,550,000 at the beginning of the LGP; in contrast, zebrafish (Fig. 3B) and Mexican tetra (Fig. 3C) trended to reach peaks approximately 0.4 mya. However, fugu (Fig. 3D), comparable with medaka (Fig. 2H), also showed *N*_*e*_ changes within a shorter period than the other species (Figs. 2 and 3).

## Discussion

Global climatic oscillations have been shown to play a vital role in the proliferation and colonization of life on our planet [37]. Five mass extinctions have been identified previously in earth’s history, primarily related to dramatic climate changes [3]. Uncovering temporal dynamics of effective population size can help better understand evolution in light of global climate variations [37]. Predictions of historical population sizes can also improve us to evaluate the impact of human activities on biodiversity [38].

### The effective use of public genome datasets in evaluating fish population sizes

The PSMC method, relying on whole-genome variation information, has been developed as an efficient approach to calculate the *N*_*e*_ value of any organism [22]. For example, Tollis et al. [39] compared the demographic difference between two species of Atlantic Humpback whales based on the PSMC calculation. They concluded that the difference between PSMC trajectories may be caused by recent admixture, intraspecific variation, and population structure. Conversely, errors generated by variations in the sequence coverage may also contribute in part to the predicted difference. Barth et al. [40] revealed similar demographic histories of Atlantic and Indo-Pacific yellowfin tuna, suggesting that the two populations diverged only recently. We and our collaborators also applied PSMC to analyze the *N*_*e*_ of the Kanglang white minnow [26], and concluded that the population demography was highly consistent within the three development periods of local habitat in the Fuxian Lake. In addition, our research group reported the first genome assemblies of amphibious fishes for three mudskipper species [23]. In this interesting study, a tight correlation between demographic expansion (or bottleneck events) and sea-level fluctuations was well established in two representative mudskippers.

Here, with public availability of high-quality genome assemblies and sequencing data (Table S1), PSMC was used to predict the temporal dynamics of several representative fish species. Our results presented here depicted multiple rounds of population contraction and expansion in most examined fish species during the Neogene and the Quaternary periods (Fig. S2). In fact, 83% (10/12) of the fish examined showed a drastic reduction in the *N*_*e*_ values before the beginning of the LGP (110–12 kya). Nearly all of the examined species had a lower *N*_*e*_ during recent periods, in comparison to the peak(s), which can be explained by increasingly dramatic glaciation over the past few million years. These results provide a comprehensive summary of the historical *N*_*e*_ changes in various fishes, which can also help us to protect endangered species amidst future global climate changes.

### The overall correlation between climate change and variations in *Ne*

Multiple rounds of *N*_*e*_ increase and decline were observed in most of the examined fish species. These observations may potentially be related to the drastic variations in global climate that occurred during the Neogene and the Quaternary periods. Over several million years, climatic glaciation movements appeared in a relatively fixed order and the cycle repeated every 100,000 years or less [32]. Consequently, fish species were forced to adapt to new environmental circumstances during interglacial periods. Climatic glaciation movements may have contributed to the severe decline in population sizes, or even in some cases of extinction. During the mild interglacial periods, however, the population sizes recovered rapidly (see more details in Fig. 1B).

In our present study, we observed a reduction pattern that appeared in most studied fishes since the beginning of the LGP. This reduction pattern was also observed in birds [24] with a slight difference at the beginning of the decline. We also observed that the overall *Ne* slowly recovered since 20 kya (Figures S2), especially in those fishes with fluctuations of effective population expansions and contractions.

During 100 kya to 1 mya, the alternating glacial and interglacial periods might have impacted on the *Ne* in several species, which live on the upper of water with warmer water temperature. For example, we observed a significant climbing of *Ne* in Nile tilapia during this period; oppositely, when experiencing the cold climate, its *Ne* declined dramatically (Fig. 2C). Nile tilapia, native to tropical West Africa, is a tropical species that prefers to live in the temperature ranging from 31 to 36 °C. It’s spawning only occurs when the water temperature reaches up to 24 °C [41]. Thus, the colder temperature contracted the *Ne* dramatically during glacial periods. This pattern was also confirmed in half-smooth tongue sole, Japanese flounder (Fig. 2D), spotted green pufferfish (Fig. 2G), stickleback (Fig. 3A) and Mexican tetra (Fig. 3C). However, during 1mya to 10 mya, no clear pattern was determined to discern the *Ne* in most of examined fishes.

### Ambiguous correlations between heterozygosity and effective population size

Whole-genome heterozygosity has been widely accepted as a measure of genetic variation in many species [42]. Heterozygosity also provides useful information of long-term *N*_*e*_ changes [43]. A high-level of heterozygosity usually represents a large *N*_*e*_ value, as only sufficiently large populations can harbor extensive genetic diversity [44]. In contrast, a low-level of heterozygosity reflects a small *N*_*e*_, as small populations may easily lose genetic diversity [42]. Based on a probabilistic model of coalescence with recombination and variations in heterozygosity over time, the PSMC algorithm can predict *N*_*e*_ values in any individual diploid species.

In our present study, an unclear relationship was drawn between heterozygosity and *N*_*e*_*.* For example, zebrafish showed the highest level of heterozygous sites among the examined species. However, its *N*_*e*_ values, whether the peak of *N*_*e*_ or the amplitude of variation, in zebrafish were not the most remarkable when compared with the other examined fishes (Fig. 3B). Zebrafish experienced only one round of *N*_*e*_ increasing and decreasing, in comparison with multiple rounds of observed fluctuations in most other species (Figs. 2 and 3).

In the practice of PSMC analysis, mutation rate estimation can be a predictor of sequence biodiversity. Purifying selection, like inbreeding and genetic drift, can lead to reduction of heterozygosity and thereby loss of heterozygous sites [45]. Practically speaking, mutations in the PSMC analysis are independent of one another. The influence of reduced heterozygosity due to selection is correlated to the results of a reduced mutation rate.

Low coverage of genomic regions can also lead to an underestimation of *N*_*e*_ [46]. For those diploid genomes with low coverage of sequencing, heterozygotes will be randomly lost due to the lack of coverage of both alleles. This also has a similar effect as the lower mutation rate [47]. In our present study, Mexican tetra and medaka with the lowest genomic coverage (86.2 and 79.3%, respectively; Supplementary Table S1) exhibited a shorter period of *N*_*e*_ (Fig. 3C and Fig. 2H).

### Drawbacks of the PSMC method

We observed a poor performance of the PSMC method when it was implemented for non-natural species. In this paper, we operated PSMC for two channel catfish that were considered with the same origin. However, the *Ne* values in the two fishes didn’t show similar patterns. Thus, the output of PSMC could become unreliable when natural species experienced artificial selection within tens of generations. During the process of artificial selection, various traits with economic values could be manually selected and then reinforced throughout numerous generations. This process indeed modifies the original genomic feature, which will destroy the PSMC analysis from the beginning stage. Therefore, when using PSMC to infer *Ne* values, we should consider whether the examined species is natural or not.

Similar to any other population genetics method for analyzing high-throughput genomic sequencing data, the PSMC is influenced by sequencing errors and missing data [46]. Genotyping sites with low coverage can lead to misidentification of heterozygous sites, which will be classified as false positives [48]. By comparing different degrees of genome-wide coverage and missing data rate in black-and-white Ficedula flycatchers, Nadachowska-Brzyska et al. [25] proposed that the appropriate genome-wide coverage should be higher than 18-fold with less than 25% of missing data for a reasonable PSMC analysis.

With the exception of the quality of sequencing data, the parameter estimation for PSMC may also play a critical role in demographic analysis, such as using the scaling parameters for an explanation of the results. Proper mutation rate (u) and generation time (g) are the prerequisites for scaling the outputs of PSMC analyses. Thus, if these two parameters are misjudged, any PSMC analysis would become tendentious. Fortunately, however, both mutation rate and generation time affect the PSMC results in a fixed manner. The principal pattern of a PSMC curve can be corrected by sliding the target curve along the axes [24]. In our present study, the divergence time estimation was accurate, which is consistent with the analyses of multiple nuclear gene sequences [49] and Fish T1K transcriptomes [50]. With the literature supported generation time (Table S2), our PSMC analysis generated trustful outputs.

Despite its wide usage, the PSMC method is restricted to a limited number of samples. This may lead to an impossible inference of recent population size history. PopSizeABC, a new Bayesian computational approach, uses a large set of samples and allows an inference to the evolution of the *N*_*e*_ over recent time [51]. In contrast to the PSMC with only reconstruction of the scenarios before 10 kya, this novel method can infer the most recent (even the last 100 generations) population size of any examined species. This method uses a small number of statistics related to allele frequencies and linkage disequilibrium. Recently, the PopSizeABC method has been well applied in four cattle breeds and in an endangered bird, the crested ibis [52].

## Conclusions

The temporal dynamics of *N*_*e*_ provide essential information for interpreting and projecting evolutionary outcomes during the global climate changes. Using high-quality genome assemblies and corresponding sequencing data, we implemented PSMC method to estimate the *Ne* changes of twelve representative teleosts from approximately 10 mya to 10 kya. Multiple rounds of population contraction and expansion in most of the examined species were observed during the Neogene and the Quaternary periods. In comparison with the peaks, almost all of the teleosts maintained long-term lower *N*_*e*_ values during the last few million years, which reflect the increasingly dramatic glaciation during this period. Our study provides a more comprehensive understanding of the historical *Ne* changes in teleosts. Results could be meaningful for the protection of species in light of ongoing global climate changes.

## Methods

### Genome data collection

For these public teleost sequencing projects, relevant genome data (Table 1) were downloaded from several public databases, including NCBI, Ensembl, and several professional databases (such as the Grass Carp Genome Project). The genome assembly and raw sequencing data of each fish species were obtained individually (see more details in Tables 1 and S1).

### Heterozygous SNP calling and heterozygosity estimation

For each species, the ALN module of Burrows-Wheeler Alignment (BWA) v0.7.12 [53] was used with default parameters to align raw sequencing data (Table S1) with the corresponding genome assembly (Table 1). Subsequently, Picard-tools v1.117 (http://broadinstitute.github.io/picard/) “SortSam” and “MarkDuplicates” were used to sort and delete duplicates in the alignment output. Then, Samtools v1.5 [54] “mpileup” module with the parameter “-C50” and the Bcftools v1.5 [54] “call” module with the parameter “-vmO v -V indels” were used to generate the raw SNP dataset. Based on the average mapping depth of reads, the minimal read depth was set to one third of the average mapping depth and the maximal depth was set to twice the average mapping depth in order to discard low-quality SNP markers. Using a local Perl script, SNPs with the distance to the next marker less than 10 bp was discarded. The final dataset of refined heterozygous SNPs was obtained for each species examined. The heterozygosity rate of each species was calculated based on the number of heterozygous SNPs and the genome size of each species.

### Mutation rate estimation

To obtain the mutation rate of each species, a divergence time tree of related species (Figure S1) was constructed. For this analysis, the protein coding sequences of teleosts were collected from reported genome assemblies (Table 1). In total, a 628 one-to-one orthologues gene set was generated from 22 species by using the Blastp (Altschul et al., 1990) and Hcluster_sg [55] with a parameter of “-w 10 -s 0.34”. The Bayesian Inference (BI) method was applied to perform phylogenomic analyses. MrMTgui [56] program was employed to complete the best-fit nucleotide substitution model test. The general time reversible (GTR) model with optimization of substitution rates and Gamma model of rate heterogeneity (GTR + I + G) were the best-fits. Finally, a phylogenetic tree was constructed using MrBayes v3.1.2 [57] with the parameter setting as “ngen = 100,000”. Branch support confidence was assessed by Bayesian posterior probabilities. Based on the topology generated from the phylogenomic analysis, the divergence time of these species was inferred by applying the Mcmctree module of PAML package [58] with the parameter “clock = 2, model = 4”. Six branch nodes were calibrated using fossil records (Figure S1).

Almost all of our divergence age estimations were accurate. We estimated that the *P. formosa* diverged with *X. maculatus* at 9.4–27.9 mya, which agrees with the analyses of multiple nuclear gene sequences [49] and Fish T1K transcriptomes [50]. We estimated the divergence time between the Ostariophysi (*D. rerio* and *I. punctatus*) and the Clupeiformes at 242.3 mya (95% interval: 211.5–272.0 mya), which is also consistent with the above-mentioned analyses. The divergence time between *I. punctatus* and *A. mexicanus* was 118 mya (95% HPD interval: 104.6–143.4 mya), which is also validated in the above-mentioned analyses. The accurate divergence time estimations (Table S2) indicated our mutation rate calculation more convincingly.

For the second step, the zebrafish genome was used as the reference to obtain whole-genome level pairwise alignments between zebrafish and other fish species using the LASTZ program v1.02.00 [59] with the parameter setting as “--step = 19 --hspthresh = 2200 --inner = 2000 --ydrop = 3400 --gappedthresh = 10,000 --format = axt”. The length of alignment block without any gaps was ascertained and Ns was counted. The number of variation sites in each alignment block was also counted. The following formula was applied to calculate the mutation rate of each fish: u = Nv/Len/2 t, where u is the mutation rate, Nv is the number of variation sites, Len is the length of the alignment block, and t is the divergence time between zebrafish and each examined species based on the phylogenomic analysis (Table S2).

### Demographic history

To predict the demographic history of each fish species, the pairwise sequentially Markovian Coalescent (PSMC) model [22] was applied with the parameter “-N 30 -t 15 -r 5 -b -p “4 + 10*1 + 20*2 + 4 + 6″“. Only those heterozygous SNP loci with minor allele frequency (MAF) ≥ 0.2 were used. Subsequently, 100 bootstraps were operated for each species to examine the variance in *N*_*e*_ estimates. Finally, the results were scaled by using the generation time and mutation rate of each species to construct the demographic history from 10 kya to 10 mya. The figures for the demographic history of each fish species were generated by gnuplot (http://www.gnuplot.info/).

## Supplementary Information

**Additional file 1: Table S1.** Statistics of the aligned sequencing data. **Table S2.** Mutation rates and generation times of the examined fish species. **Table S3.** The SRA data of the examined fish species used in this study. **Figure S1.** The divergence time tree of 22 representative species. Tropical clawed frog (*X. tropicalis*) was used as the outgroup. The branch nodes with crimson dots were calibrated by using reported fossil records. **Figure S2.** Historical *Ne* in different aspects.

## Data Availability

Raw sequencing and genome data were previously published by different studies. The repository information for each species can be found in Table 1 and Supplementary Table S3. All datasets generated by analyses during this study are additionally available from the corresponding author on reasonable request.

## Abbreviations

BI: Bayesian Inference
BWA: Burrows-Wheeler Alignment
*g*: Generation time
GTR: General time reversible
kya: Thousand years ago
LGP: Last glacial period
MAF: Minor allele frequency
mya: Million years ago
*N*_*e*_: Effective population sizes
PSMC: Pairwise Sequentially Markovian Coalescent
TMRCA: Time of the most recent common ancestor
*u*: Mutation rate
