# Supplementary material for: Temporal dynamics of teleost populations during the Pleistocene: a report from publicly available genome data
Source: BMC Genomics. 2021 Jun 30;22:490. doi: 10.1186/s12864-021-07816-7 (PMC8247217; doi:10.1186/s12864-021-07816-7)
Supplement: Supplementary file 1 — Additional file 1: Table S1. Statistics of the aligned sequencing data. Table S2. Mutation rates and generation times of the examined fish species. Table S3. The SRA data of the examined fish species used in this study. Figure S1. The divergence time tree of 22 representative species. Tropical clawed frog (X. tropicalis) was used as the outgroup. The branch nodes with crimson dots were calibrated by using reported fossil records. Figure S2. Historical Ne in different aspects. [file 12864_2021_7816_MOESM1_ESM.docx]

**Supplementary Table S1** Statistics of the aligned sequencing data.

| **Species** | **Raw data (Gb)** | **Sequencing depth (×)** | **Mapped data (Gb)** | **Mapped ratio (%)** | **Genome coverage** |
| --- | --- | --- | --- | --- | --- |
| Nile tilapia | 18.78 | 18.67 | 11.79 | 63.24 | 0.987 |
| Pufferfish | 112.13 | 291.93 | 53.81 | 47.99 | 0.985 |
| Red arowana | 52.14 | 69.25 | 46.65 | 89.79 | 0.982 |
| Atlantic herring | 32.38 | 44.62 | 28.14 | 86.89 | 0.981 |
| Green arowana | 46.24 | 47.70 | 62.85 | 90.73 | 0.981 |
| Japanese flounder | 108.82 | 199.39 | 106.09 | 97.41 | 0.977 |
| Stickleback | 72.95 | 158.07 | 23.92 | 32.79 | 0.958 |
| Channel catfish BGI | 115.26 | 163.57 | 90.74 | 78.72 | 0.956 |
| Golden arowana | 47.70 | 62.85 | 42.94 | 90.73 | 0.954 |
| Half-smooth tongue sole | 39.56 | 88.86 | 31.81 | 80.40 | 0.945 |
| Blue tilapia | 13.53 | 14.63 | 11.86 | 87.71 | 0.912 |
| Mexican tetra | 22.02 | 16.49 | 15.80 | 71.92 | 0.862 |
| Medaka | 25.69 | 31.68 | 22.12 | 86.10 | 0.793 |

**Supplementary Table S2** Mutation rates and generation times of the examined fish species.

| **Species** | **Mutation rate** | **Generation time** | **Reference** |
| --- | --- | --- | --- |
| Nile tilapia | 6.18e-10 | 1 | [1] |
| Pufferfish | 2.46e-09 | 4 | [2] |
| Red arowana | 4.41e-09 | 8 | - |
| Atlantic herring | 3.75e-09 | 6 | [3] |
| Green arowana | 4.41e-09 | 8 | - |
| Japanese flounder | 1.85e-09 | 3 | [4] |
| Stickleback | 1.21e-09 | 1 | [5] |
| Channel catfish BGI | 5.22e-09 | 7 | [6] |
| Golden arowana | 4.41e-09 | 8 | - |
| Half-smooth tongue sole | 1.71e-09 | 3 | [7] |
| Blue tilapia | 4.91e-10 | 1 | [8] |
| Mexican tetra | 8.70e-10 | 1 | [9] |
| Medaka | 6.18e-10 | 1 | [10] |

**Supplementary Table S3** The SRA data of the examined fish species used in this study.

| **Species** | **Bioproject** | **SRA** | **Insert size (bp)** |
| --- | --- | --- | --- |
| Nile tilapia | PRJNA59571 | SRR071591  SRR071597  SRR071603  SRR071612 | 420  420  420  419 |
| Red arowana | PRJNA290062 | - | - |
| Atlantic herring | - | SRR1943163  SRR1945056  SRR1945057 | 500  500  800 |
| Green arowana | PRJNA290061 | - | - |
| Japanese flounder | PRJNA73673 | - | - |
| Stickleback | PRJNA525775 | SRR9087171  SRR9087174  SRR9087175  SRR9087180 | 800  800  800  800 |
| Channel catfish BGI | PRJNA319455 | SRR3497286  SRR3497291  SRR3497292 | 250  500  800 |
| Golden arowana | PRJNA290065 | SRR3405401  SRR3405402 | 500  800 |
| Half-smooth tongue sole | PRJNA73987 | SRR351291  SRR351292  SRR351293  SRR351294  SRR351305  SRR351309  SRR351313 | 785  785  502  502  500  495  750 |
| Blue tilapia | PRJNA539829 | - | - |
| Mexican tetra | PRJNA533584 | SRR608903  SRR608904  SRR608905  SRR608908 | 340  346  573  340 |
| Medaka | PRJEB2759 | ERR110349  ERR110350  ERR110351  ERR110352 | 497  497  496  496 |

**Reference**:

1. Duponchelle F, Panfili JJEBoF. Variations in age and size at maturity of female Nile tilapia, *Oreochromis niloticus*, populations from man-made lakes of Côte d'Ivoire. 1998;52(4):453-65.

2. Ogawa K, Inouye KJFP. Heterobothrium infection of cultured tiger puffer, *Takifugu rubripes*—A field observation. 1997;32(1):15-20.

3. Feng C, Pettersson M, Lamichhaney S, Rubin C-J, Rafati N, Casini M, et al. Moderate nucleotide diversity in the Atlantic herring is associated with a low mutation rate. 2017;6:e23907.

4. YONEDA M, KURITA Y, KITAGAWA D, ITO M, TOMIYAMA T, GOTO T, et al. Age validation and growth variability of Japanese flounder Paralichthys olivaceus off the Pacific coast of northern Japan. 2007;73(3):585-92. doi: 10.1111/j.1444-2906.2007.01371.x.

5. Ghani NIA, Herczeg G, Leinonen T, Merilä J. Evidence for genetic differentiation in timing of maturation among nine-spined stickleback populations. 2013;26(4):775-82. doi: 10.1111/jeb.12085.

6. Liu Z, Liu S, Yao J, Bao L, Zhang J, Li Y, et al. The channel catfish genome sequence provides insights into the evolution of scale formation in teleosts. 2016;7(1):1-13.

7. Jing-feng Y, CHEN S-l, ZHAI J-m, TIAN Y-s, SU P-z, SUN L-jJJoIMUfN. Artificial Propagation of Half-smooth Tongue-sole, *Cynoglossus Semilaevis*. 2010;(2):27.

8. Maclean N, Rahman M, Sohm F, Hwang G, Iyengar A, Ayad H, et al. Transgenic tilapia and the tilapia genome. 2002;295(2):265-77.

9. Jeffery WRJDb. Cavefish as a model system in evolutionary developmental biology. 2001;231(1):1-12.

10. Ozato K, Wakamatsu Y, Inoue KJMmb, biotechnology. Medaka as a model of transgenic fish. 1992;1(4-5):346.

**
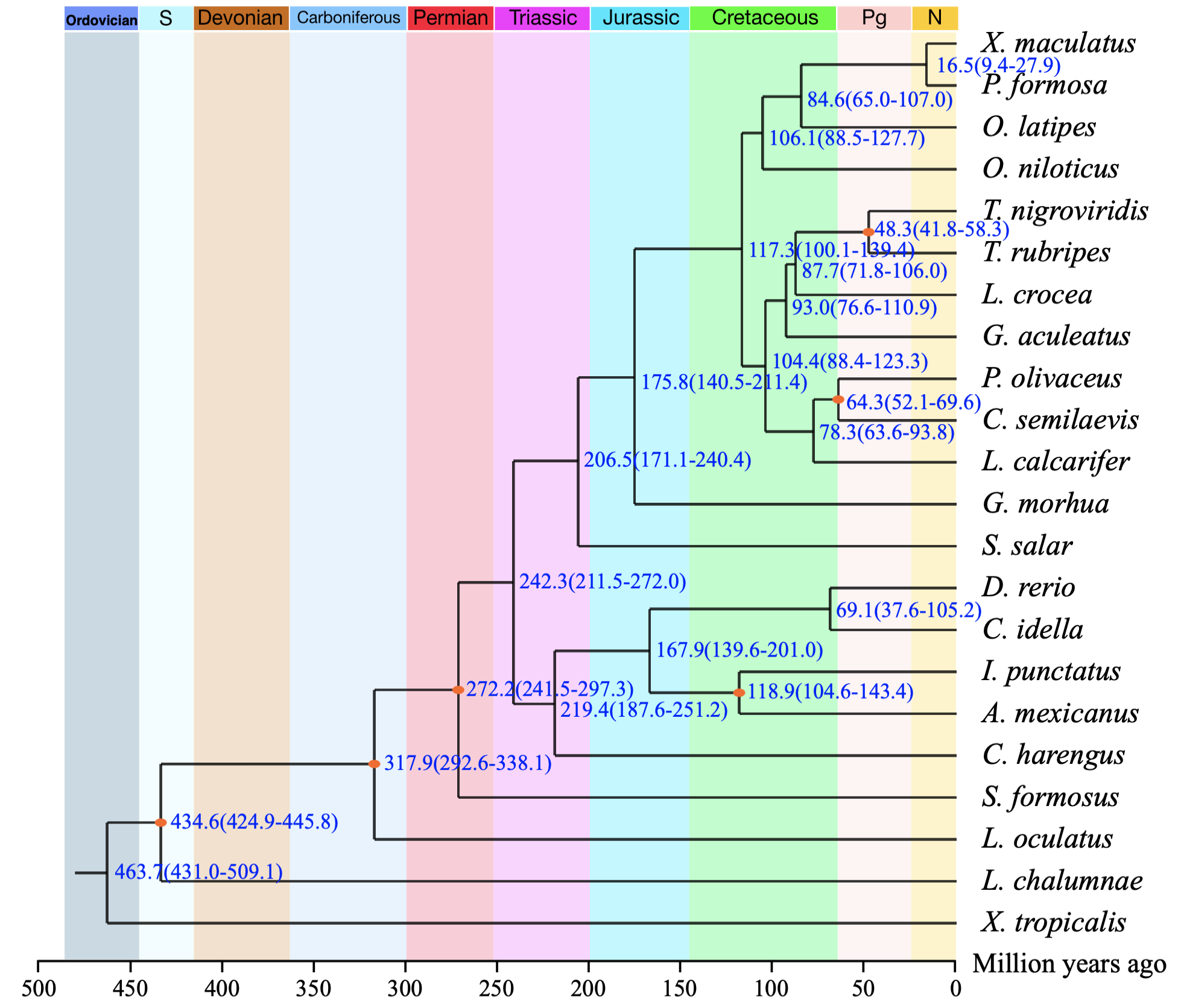
**

**Supplementary Figure S1 The divergence time tree of 22 representative species.** Tropical clawed frog (*X. tropicalis*) was used as the outgroup. The branch nodes with crimson dots were calibrated by using reported fossil records.


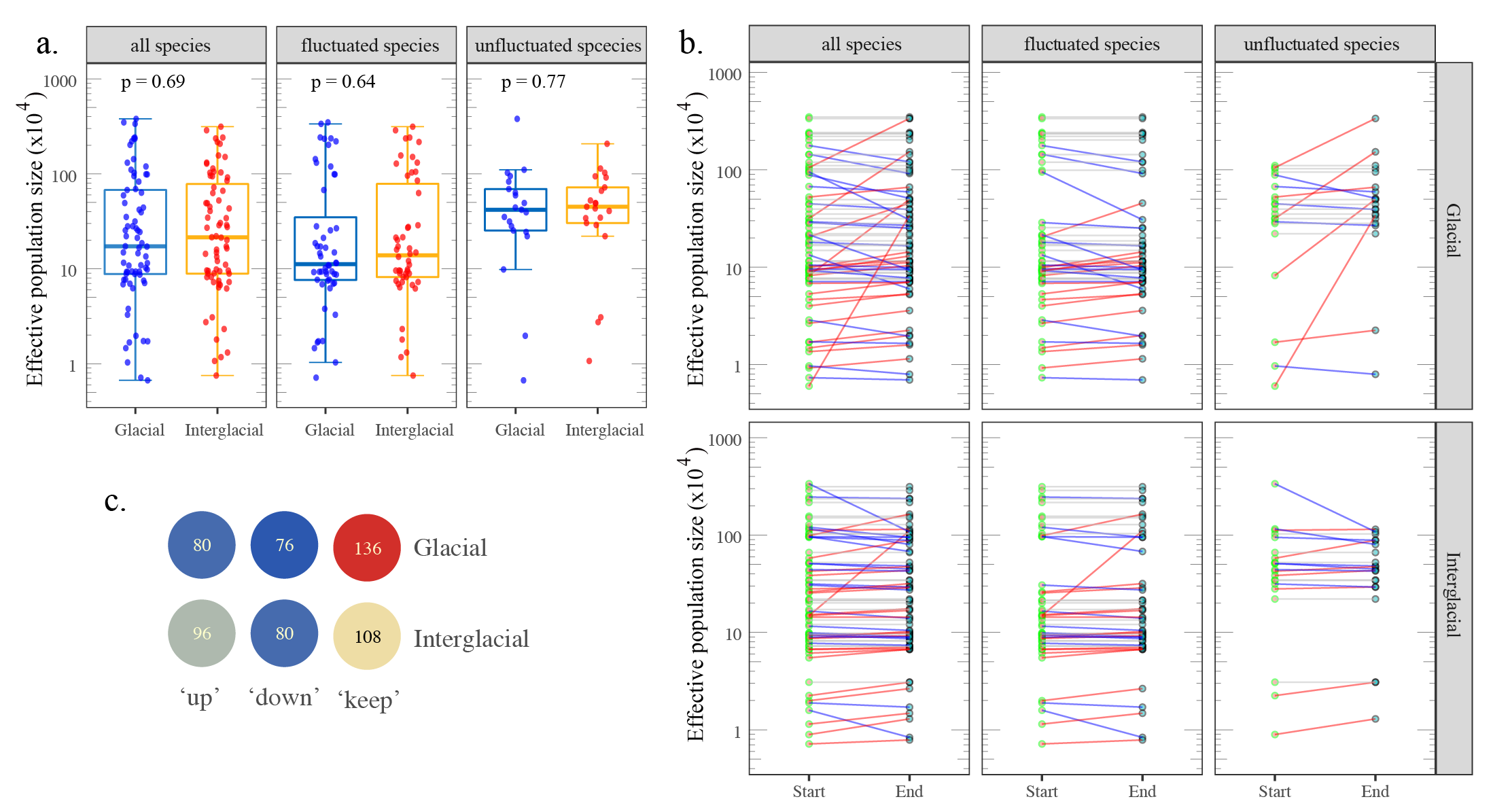


**Supplementary Figure S2 Historical *Ne* in different aspects.** **a**) The average *Ne* of each glacial/interglacial period of each species. The “fluctuated species” means those species with fluctuations of effective population expansions and contractions. These species also were used to generate Figures 2 and S3. The “unfluctuated species” mean those species without fluctuations of *Ne*. These species were also used for generation of Figures 3 and S4. **b**). The variation tendency of *Ne* of each glacial/interglacial period in each species. The *Ne* at the starting time (Start) and ending time (End) of each period in every species had been used for plotting the tendency. **c**). The number of three different tendencies in b) was counted. “up” means the rising trend in b) with red lines; “down” means the descending trend in b) with blue lines; “keep” means the *Ne* without significant change (grey lines in subfigure b).
